# Acute appendicitis: a case report of hyperinfection with *Enterobius vermicularis*

**Published:** 2021

**Authors:** Hossein Hooshyar, Mohammad Jannati Dastgerdi, Ebrahim Kazemi

**Affiliations:** 1 *Department of Parasitology and Mycology School of Medicine. Kashan University of Medical Sciences. Kashan-Iran.*; 2 *Faculty of Medical Sciences and Health Services Khoy, Urmia University of Medical Sciences, Urmia, Iran *

**Keywords:** Appendicitis, Parasite, Enterobius, Khoy

## Abstract

Acute appendicitis is one of the most common causes of abdominal emergent surgical disease worldwide. *Enterobius vermicularis,* a human intestinal parasite, is reported to be associated with acute appendicitis.

We report a case of an 8-year-old girl who was admitted to the emergency unit with complaints of severe abdominal pain and was diagnosed with acute appendicitis. Microscopic pathological examination showed lymphoid follicles with prominent germinal centers and mantle zones within the appendix wall. Cross-sections of multiple female and male *Enterobius vermicularis* worms and a few longitudinal sections of *E.vermicularis *were seen.

*E. vermicularis* is one of the most common human parasitic infections, so the possibility of infection of the appendix with *E. vermicularis *should be considered in the differential diagnosis of agents of appendicitis.

## Introduction

 Acute appendicitis is one of the most common causes of abdominal emergent surgical disease worldwide (1). The lifetime risk of acute appendicitis is reported to be approximately 8.6% for males and 6.7% for females (1-2), and it is more prevalent in children and adolescents. The peak of the incidence of this condition occurs in the second-decade of life (3).

Appendectomy is the choice treatment for acute appendicitis. The mortality rate after appendicitis surgery is very low and reported to be from 0.07% to 0.7% and 0.5% to 2.4% in patients without and with perforation, respectively (4). Little is known about the cause of acute appendicitis, but it has been well established that the primary cause of appendicitis is probably intraluminal obstruction, which may result from lymphoid hyperplasia, parasite-associated infection, fecal matter, ingested foreign bodies, or primary neoplasms or metastasis (5-6). Among the mentioned causes, foreign bodies, parasitic helminths and protozoa, upper respiratory infection, mononucleosis, Crohn’s disease, and primary or metastasis cancer have been reported to rarely develop acute appendicitis (6-7).

The relation between parasitic infections and acute appendicitis has been the subject of discussions for many years (7-8). Some human intestinal helminths and protozoan parasites such as *Enterobius vermicularis, Ascaris lumbricoides, Trichuris trichura, Entamoeba histolytica*, *Taenia spp*., and, very rarely, *Balantidium coli *are reported to be associated with acute appendicitis (9). Among the parasitic agents, *Enterobius vermicularis* is considered as the most common helminthic infection found in the lumen of the appendix and related to acute appendicitis (8-9). The presence of *E. vermicularis* in the lumen of the human appendix was reported for the first time by Fabrius in 1634 (10).

Enterobiasis is one of the most common parasitic diseases in Iran and worldwide. A meta-analysis study showed that approximately 17% of kindergarten and primary school children in Iran were infected with *E. vermicularis *(11).

Ramezani and Dehghani reviewed 5,048 appendix specimens delivered to the Department of Pathology at the two teaching hospitals in Kerman during the 10- year period from 1993 to 2003, and found *E. vermicularis* in 144 (2.9%) cases (8). 

A retrospective study on 1533 removed appendices at Al-Zahra Medical Center in Isfahan, Iran, revealed that five tissue specimens (0.3%) had *E. vermicularis *infections during 2001-2006 (12). 

In the present study, a case of acute appendicitis who underwent an appendectomy is described. The pathology results for this patient revealed multiple cross sections and a number of *E. vermicularis eggs*. 

## Case Report

**Figure 1:**
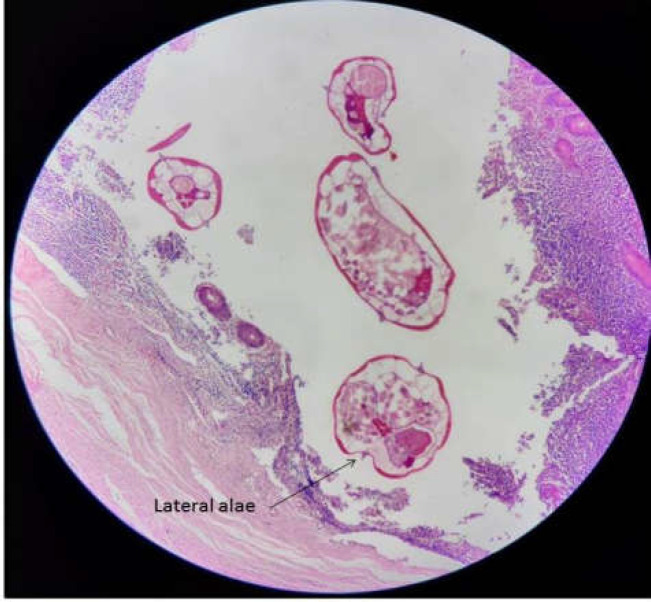
Cross-section of multiple female and male *Enterobius vermicularis* worms in appendix lumen

**Figure 2:**
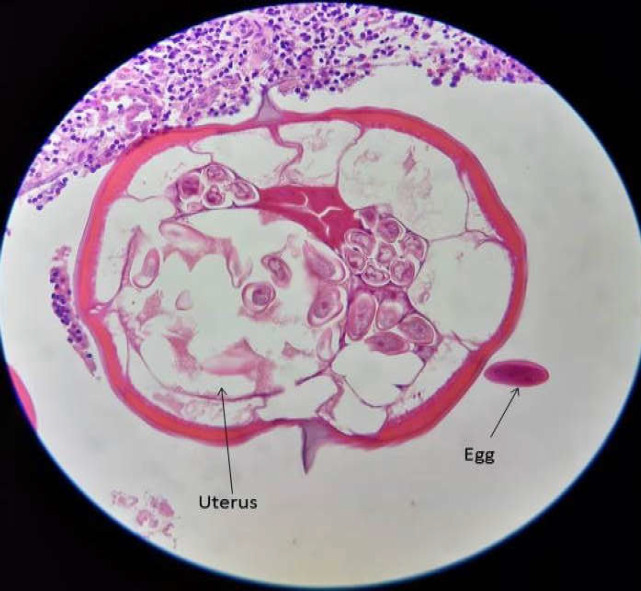
Cross-section of female worm with free egg in appendix lumen

**Figure 3:**
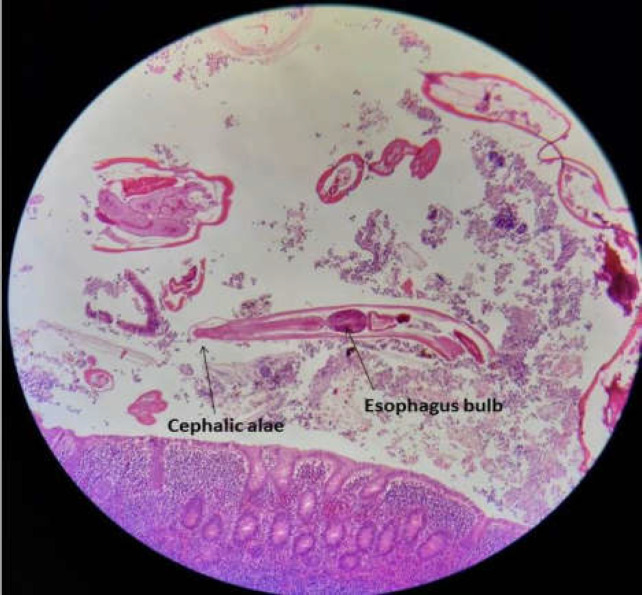
Cross-section of multiple female and male *Enterobius vermicularis* and longitudinal section of worm with typical cephalic alae and muscular posterior bulb of esophagus in appendix lumen

An 8-year-old girl was admitted to the emergency unit and hospitalized in the Surgical Outpatient Department of Ghamar Bani Hashem Hospital, Khoy, West Azarbaijan Province, in May 2020 with complaints of severe pain in the right lower abdomen, nausea, vomiting, anorexia, but no fever. She was diagnosed with acute appendicitis. Other physical system examinations of the patient were normal, and the patient’s vital signs were stable. Chest X-ray was also normal. An abdominal ultrasound revealed the presence of an inflamed appendix. The patient did not show any significant findings in her present and past medical history. Laboratory tests of the patient were within normal limits except for a higher WBC count (12400/mm3). An open appendectomy was performed. The appendix was edematous and non-perforated. In macroscopic findings, the specimen was received in formalin and measured 3 cm in length and 0.4 cm in diameter. The appendix specimen was embedded in paraffin, and some tissue sections were prepared and stained with Haematoxylin-Eosin (H&E). Microscopic pathological examination showed lymphoid follicles with prominent germinal centers and mantle zones within the appendix wall. The most observed pathologic finding was also reactive follicular hyperplasia. Cross-sections of multiple female and male *E. vermicularis *worms with some free eggs of worms (Figs. 1, 2) and a few longitudinal sections of *E. vermicularis *with typical cephalic alae and a muscular posterior bulb of the esophagus were seen (Fig. 3).

The patient’s symptoms improved on the second postoperative day, and she was discharged with recovery.

## Discussion

Among the numerous causes of appendicitis, parasitic infections, especially *E. vermicularis, *is one. A recent meta-analysis study showed that the overall prevalence of *E. vermicularis* in cases of appendicitis identified by histopathological methods is estimated to be 4% (13). Abdellatif et al. showed that 3% of surgically removed appendices were positive for *E. vermicularis* in El Minia, Egypt (6). In two studies in different regions of Iran, *E. vermicularis *was identified in 2.9% and 0.3% of surgically removed appendices from Iranian populations (6, 12).

The results of the present study showed several male and female worm sections with a number of eggs in most of the slides, whereas in most similar studies, only one or two cross sections of *E.vermicularis *were detected (12, 14).

Most studies on histopathological variations of helminthic acute appendicitis have shown a relatively high frequency of neutrophil infiltration and purulent exudate as the most common findings (14-15). In the present case, the most observed pathological finding was reactive follicular hyperplasia with no sign of inflammation. In most previously reported cases, it seems that *E. vermicularis* had caused reactive follicular hyperplasia (12-14).

*E. vermicularis* can cause inflammation and thus, appendicitis. Inflammation of the appendix may develop if *E. vermicularis* or parasite ova block the lumen.

The present case was an 8-year-old girl. It has been well established that acute appendicitis is more prevalent in children and adolescents (3). Age is a risk factor for acute appendicitis, and the interaction of age with *Enterobius* infection in both sexes has been determined (8, 13).

We report the case of an 8-year–old girl with acute appendicitis due to hyperinfection with *Enterobius vermicularis*. It can be concluded that *E. vermicularis* is one of the most common parasitic infections that may increase the risk of appendicitis in young children. Therefore, the possibility of infection of the appendix with *E. vermicularis* should be considered in the differential diagnosis of agents of appendicitis.

Complete treatments and control strategies of helminthic-infected children may influence the prevention of serious complications such as appendicitis.
